# Cardiotoxic Effects of Osimertinib Compared to Other EGFR Inhibitors: A Systematic Review and Meta-Analysis

**DOI:** 10.1007/s12012-026-10106-x

**Published:** 2026-03-09

**Authors:** Alan Garcia, Abdul Mueez Alam Kayani, Daniel Alejandro Navarro-Martinez, Ricky E. Lemus-Zamora, Richard Salama-Frisbie, Thomas Fretz, Eduardo Tellez-Garcia, Eduardo Aviles, Brijesh Patel

**Affiliations:** 1 Department of Internal Medicine, Indiana University School of Medicine, 340 West 10th Street Fairbanks Hall, Suite 6200, Indianapolis, IN 46202-3082 USA; 2 Department of Internal Medicine, AdventHealth Tampa, 3100 E Fletcher Ave, Tampa, FL 33613 USA; 3 Department of Internal Medicine, Instituto Nacional de ciencias Médicas y Nutrición Salvador Subirán, CDMX, Vasco de Quiroga 15, Belisario Domínguez Secc 16, Tlalpan, Ciudad de México, 14080 México; 4 Department of Cardiovascular Medicine, Indiana University School of Medicine, 340 West 10th Street Fairbanks Hall, Suite 6200, Indianapolis, IN 46202-3082 USA

**Keywords:** Carcinoma, Non-small-cell lung, Receptor, Epidermal growth factor/tyrosine kinase inhibitors, Osimertinib, Cardiotoxicity, Heart failure, Myocardial infarction

## Abstract

Osimertinib, a third-generation epidermal growth factor receptor (EGFR) inhibitor, was developed to overcome resistance from EGFR-mutant non–small-cell lung cancer (NSCLC). While it offers significant therapeutic benefits, reports have linked Osimertinib to cardiotoxic effects. This study aims to clarify the direct cardiotoxicity of Osimertinib by reviewing clinical trials and cohort studies involving Osimertinib monotherapy compared to other EGFR inhibitors. A search was conducted in online databases. Measured outcomes included risk of heart failure (HF), myocardial infarction (MI), decline in left ventricular ejection fraction (LVEF), arrhythmias, and pericardial effusion. These outcomes were reported as risk ratio (RR) with a random effects model using 95% confidence intervals (CI). Five studies with 19,008 patients (age 68 ± 13, 65% female) were selected. Osimertinib therapy was associated with an increased risk of HF (RR = 1.45, 95% CI 1.19–1.76, *p* = 0.0002), decline in LVEF (RR = 3.10, 95% CI 1.72–5.59, *p* = 0.0002) and MI (RR = 1.40, 95% CI 1.09–1.79, *p* = 0.0078) compared to other EGFR inhibitors. There was no difference in the risk of arrhythmias and pericardial effusion. Osimertinib therapy is associated with an increased risk of HF and a decline in LVEF compared to other EGFR inhibitors, while associations with MI and arrhythmias were less consistent. Although these events are infrequent, their potential severity warrants proactive cardiac monitoring for patients receiving Osimertinib, particularly in patients with pre-existing risk factors.

**Supplementary Information:**

The online version contains supplementary material available at 10.1007/s12012-026-10106-x.

## Introduction

Targeted therapies have changed the therapeutic approach to treat non–small–cell lung cancer (NSCLC). Although NSCLC encompasses tumors with similar histopathologic classification, these malignancies occur secondary to a diverse array of oncogenic alterations. Common driver mutations and rearrangements occur in genes such as *KRAS*, *EGFR*, *ALK*, *ROS1*, *BRAF*, and *NTRK1* [1].

A broad spectrum of small-molecule inhibitors has been developed to selectively target these key oncogenic drivers, leading to the approval of several agents by the U.S. Food and Drug Administration. Of these medications, the epidermal growth factor receptor (EGFR) inhibitors were among the first to enter clinical use and remain central to the precision medicine approach in NSCLC [2]. First-generation EGFR tyrosine kinase inhibitors (TKIs), such as Erlotinib and Gefitinib, demonstrated clinical efficacy but were frequently limited by dermatologic and gastrointestinal toxicities [3]. Resistance to these agents is commonly mediated by the emergence of the EGFR T790M gatekeeper mutation, which increases ATP affinity and impairs drug binding through steric hindrance [4]. This mutation also confers resistance to second-generation EGFR TKIs, including Afatinib and Dacomitinib [5].

To overcome T790M-mediated resistance, third-generation EGFR inhibitors such as Osimertinib were developed. Osimertinib was designed to selectively target EGFR mutations and lung cancers with T790M resistance mutation while sparing the wild-type EGFR mutations, thereby enhancing both efficacy and tolerability [6]. Initially, Osimertinib was employed as a sequential therapy in patients who acquired the T790M mutation during treatment with earlier-generation TKIs. However, recent clinical trials, including the AURA3, FLAURA and MARIPOSA trials, have demonstrated the superiority of Osimertinib in treatment naive patients with EGFR-mutated stage IIIB or IV NSCLC, showing significant improvements in both progression-free and overall survival [6–9].

Despite the therapeutic benefits of Osimertinib, data have demonstrated a correlation with cardiotoxic effects, including QTc prolongation, heart failure (HF), and a decrease in left ventricular ejection fraction (LVEF). The mechanisms underlying these adverse events are multifactorial and not yet fully elucidated [7].

One proposed mechanism involves off-target effects on the ErbB/HER family of tyrosine kinase receptors, particularly human epidermal growth factor receptor 2 (HER2). HER2 signaling is essential for cardiac development and the maintenance of myocardial structure and function, especially under hemodynamic stress [10]. Therapeutic agents that target HER2, such as Trastuzumab, are known to carry a risk of cardiotoxicity. While the resulting cardiac dysfunction is typically reversible and not dose-dependent, irreversible damage has also been reported in some cases [11].

Preclinical studies have shown that Osimertinib and its active metabolite AZ5104 can inhibit HER2 phosphorylation. This inhibition can impair a critical pathway for cardiomyocyte survival and contraction called neuregulin-1/HER2/HER4. Such disruptions mirror the cardiotoxic effects observed with Trastuzumab therapy [12]. Moreover, Osimertinib may interfere with additional cardiac pathways, including the phosphoinositide 3-kinase / protein kinase B (PI3K/AKT) pathway, which plays a vital role in cardiomyocyte homeostasis. Inhibition of these survival pathways may contribute to HF and other forms of cardiac dysfunction [13].

While the data linking HER2-targeted therapies to HF remains variable, recent clinical reports provide growing evidence to support the mechanistic plausibility of Osimertinib-induced cardiotoxicity [14]. Nonetheless, the specific attribution of Osimertinib cardiotoxic side effects remains challenging due to heterogeneous treatment regimens that frequently involve multiple concurrent therapies [15].

In addition to drug-specific mechanisms, traditional cardiovascular risk factors such as hypertension, diabetes mellitus, and smoking are well-recognized contributors to baseline cardiovascular dysfunction, these factors may influence susceptibility to TKI-related cardiotoxicity, possibly through endothelial dysfunction, systemic inflammation, and reduced cardiac reserve [16]. Emerging clinical data in NSCLC patients treated with Osimertinib further suggest that a history of smoking is independently associated with a higher incidence of cardiotoxicity, underscoring the relevance of traditional risk factors in this context [17].

The aim of this study is to conduct a systematic review and meta-analysis to compare the risk of cardiotoxicity associated with Osimertinib therapy versus other EGFR inhibitors in lung cancer.

## Methods

This meta-analysis was conducted per the Preferred Reporting Items for Systematic Review and Meta-Analyses (PRISMA) and A Measurement Tool to Assess Systematic Reviews 2 (AMSTAR 2) guidelines [18, 19]. The checklists for PRISMA 2020 and AMSTAR 2 guidelines are shown in Supplemental S1 and Supplemental S2. The study protocol was registered in PROSPERO (ID #CRD420251083202). An institutional review board and informed patient consent for study participation was not required, as this study was a systematic review and meta-analysis of previously published studies.

A meta-analytic approach was used to compare the cardiotoxic profile of Osimertinib to other EGFR tyrosine kinase inhibitors (TKIs) in the treatment of NSCLC with positive EGFR mutations. A comprehensive literature search was conducted in PubMed, Web of Science, and Cochrane CENTRAL, covering studies published from the inception of these databases to December 2025. Complete search strategy is shown in Supplemental S3. Additional sources were identified through manual screening of reference lists from relevant reviews and primary studies. Studies were included if they involved adult patients with EGFR-mutations in NSCLC and reported cardiovascular outcomes associated with Osimertinib therapy compared to other EGFR inhibitors. These studies comprised randomized controlled trials (RCTs), prospective cohort studies, and retrospective cohort studies. Exclusion criteria included non-peer reviewed studies, non-human studies, case reports, review articles, studies without extractable cardiovascular data, or those involving Osimertinib being used in combination regimens. Two investigators (A.G. and A.M.A.K.) independently screened eligible studies and extracted outcomes, and conflicts were resolved in consultation with a third independent reviewer (D.A.N.M.). The baseline characteristics and study outcomes data were extracted directly from the published articles and supplemental files. Outcomes to be assessed include HF, decline in LVEF, myocardial infarction (MI), arrhythmias, and pericardial effusion.

Two reviewers (A.G. and A.M.A.K.) independently assessed the risk of bias for all included studies. Discrepancies were resolved by consensus or by consulting with a third reviewer. RCTs were evaluated using the Cochrane Risk of Bias 2 (RoB 2) tool, which assesses five domains: randomization process, deviations from intended interventions, missing outcome data, measurement of the outcome, and selection of the reported result [20]. Non-randomized studies were assessed using the ROBINS-I (Risk of Bias in Non-randomized Studies of Interventions) tool, which evaluates seven domains, including confounding, selection of participants, classification of interventions, deviations from intended interventions, missing data, measurement of outcomes, and selection of the reported result [21]. Nonrandomized studies were rated as low, moderate, serious, and critical risk using the ROBINS-I tool. RCTs were rated as low risk, some concerns, and high risk of bias using the RoB2 tool. Risk of bias assessment plots were visualized through robvis tool [22].

### Statistical Methods

Statistical analyses were conducted using R (version 4.5.2), utilizing the meta package. Outcomes were reported as risk ratio (RR) with 95% confidence intervals (CIs) using the inverse variance method. A p-value < 0.05 was considered statistically significant. Heterogeneity between reported outcomes was assessed using Higgins I^2^. I^2^ statistic values < 25%, 25–50%, and > 50% were considered low, moderate, and high heterogeneity, respectively. Sensitivity analysis using the leave-one-out approach was planned for results with high heterogeneity. In addition, the influence of large observational studies on pooled estimates was assessed by pre-specified exclusion of individual studies. To explore potential sources of heterogeneity, meta-regression models were constructed with covariates including age, female sex, smoker, adenocarcinoma, White and Asian race for outcomes with at least five studies. Publication bias assessment was planned through visual assessment of funnel plots, with cautious interpretation of outcomes with fewer than 10 studies consistent with Cochrane recommendations [23].

## Results

**Fig. 1:**
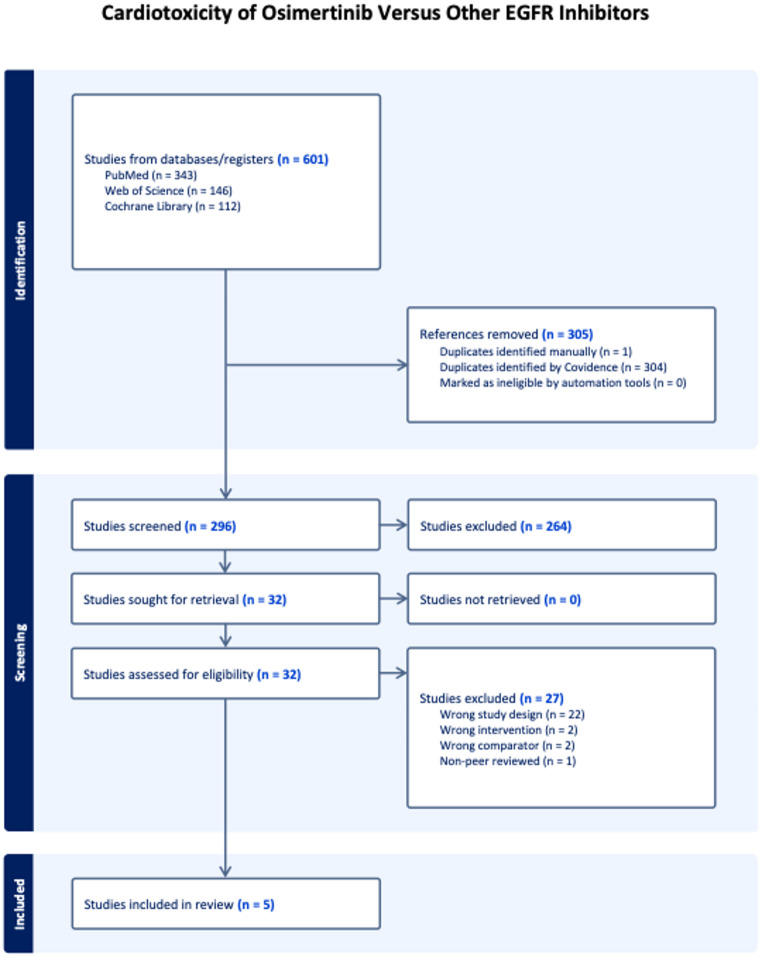
Preferred reporting items for systematic review and meta-analyses (PRISMA) flow of the search strategy for systematic review and meta-analysis

A total of 601 studies were initially identified. After screening and removal of duplicates, five studies were included in the meta-analysis with a sample size of 19,008 patients. The patients had a mean age of 68 ± 13 years, with 65% of the included population comprising females. The PRISMA flow diagram summarizes the search strategy (Fig. 1). Our meta-analysis included 9,483 patients who received Osimertinib therapy and 9525 patients who received other EGFR inhibitors. The baseline characteristics of the patients are presented in Table 1, and the follow-up period for the included studies ranged from 18 to 60 months.

**Table 1:** Baseline characteristics of patients receiving Osimertinib therapy versus other EGFR inhibitors

Author	Year	Region	Design	Database	Time Period	Follow- Up (months)	Arms	Age	N	Female	HTN	HLD	DM	Smoker	Adenocarcinoma	Brain Metastases	EGFR Mutation	
																	Exon 19 Deletion	L858R
Soria et al. - Flaura Trial [8]	2017	Multicenter	RCT	Clinical trial	Dec 2014 - Mar 2016	18	Osimertinib	62 ± 20	279	178	NS	NS	NS	97	275	53	175	104
							Gef and Erl	64 ± 17	277	172	NS	NS	NS	102	272	63	174	103
Cho et al. - MARIPOSA Trial [9]	2024	Multicenter	RCT	Clinical trial	Nov 2020 - May 2022	22	Osimertinib	61 ± 17	429	251	NS	NS	NS	134	415	172	257	172
							Ami-Laz	60 ± 18	429	275	NS	NS	NS	130	417	178	258	172
Byun et al. [40]	2024	USA	Retrospective Cohort Study	SEER-Medicare	2016–2019	36	Osimertinib	76 ± 11	1249	876	NS	NS	NS	80	1200	NS	NS	NS
							Gef, Erl and Afa	77 ± 8	1282	863	NS	NS	NS	88	1179	NS	NS	NS
Lin et al. [25]	2024	Taiwan	Retrospective Cohort Study	NCKUH (Taiwan) - EMR	Sep 2019 - July 2022	23	Osimertinib	69 ± 12	195	125	79	36	39	35	NS	NS	NS	NS
							Gef, Erl, Afa and Dac	69 ± 11	206	132	86	40	43	37	NS	NS	NS	NS
Muhanna et al. [41]	2025	USA	Retrospective Cohort Study	TriNetX	NS	60	Osimertinib	67 ± 12	7331	4731	3337	2574	1271	564	NS	1917	NS	NS
							Gef, Erl, Afa and Dac	66 ± 12	7331	4651	3376	2649	1311	609	NS	1950	NS	NS

*N* Number of Participants, *HTN* Hypertension, *HLD* Hyperlipidemia, EGFR Epidermal Growth Factor Receptor, *RCT* Randomized Controlled Trial, *Gef* Gefitinib, Erl Erlotinib, *Ami–Laz* Amivantamab–Lazertinib, *Afa* Afatinib, *Dac* Dacomitinib, *NS* Not Specified, *NCKUH* National Cheng Kung University Hospital, *EMR* Electronic Medical Record, *SEER* Surveillance, Epidemiology, and End Results

### Cardiotoxicity Results

**Fig. 2:**
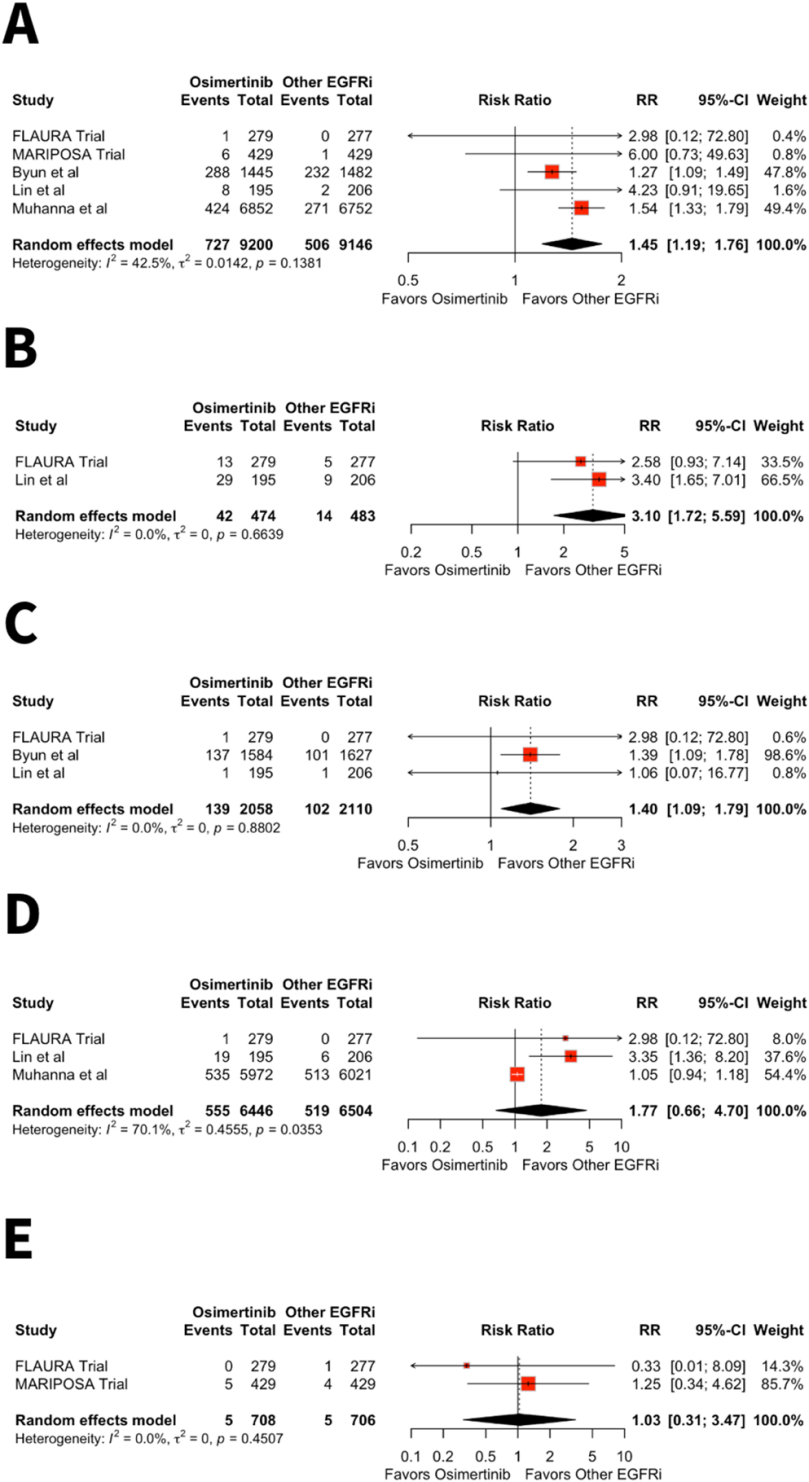
**A**-**E** Forest plots of HF **A**, decline in LVEF **B**, MI **C**, arrhythmias **D** and pericardial effusion **E** in patients receiving Osimertinib therapy compared to other EGFR inhibitors

Osimertinib therapy was associated with a significantly higher risk of HF (RR 1.45, 95% CI: 1.19–1.76, *p* = 0.0002, I² = 42.5%) (Fig. 2A), decline in LVEF (RR 3.10, 95% CI: 1.72–5.59, *p* = 0.0002, I² = 0%) (Fig. 2B) and MI (RR 1.40, 95% CI 1.09–1.79, *p* = 0.0078, I² = 0%) (Fig. 2C) compared to other EGFR inhibitors. However, there was no significant difference between Osimertinib and other EGFR inhibitors for the risk of arrhythmias (RR 1.77, 95% CI 0.66–4.70, *p* = 0.26, I² = 70.1%) (Fig. 2D) and pericardial effusion (RR 1.03, 95% CI: 0.31–3.47, *p* = 0.96, I² = 0%) (Fig. 2E).

### Bias Assessment

All the included RCTs were rated as low risk on the RoB2 tool. Observational studies were rated as moderate risk of bias on the ROBINS-I tool, given the potential for residual confounding, misclassification of outcomes, and limited adjustment for unmeasured cardiovascular risk factors. The results of risk of bias assessment using RoB2 and ROBINS-I tools are summarized in Supplemental S4 and Supplemental S5. Visual assessment of funnel plot for HF revealed mild asymmetry, raising the possibility of small study effects or publications bias (Supplemental S6).

### Sensitivity and Heterogeneity Analyses

**Fig. 3:**
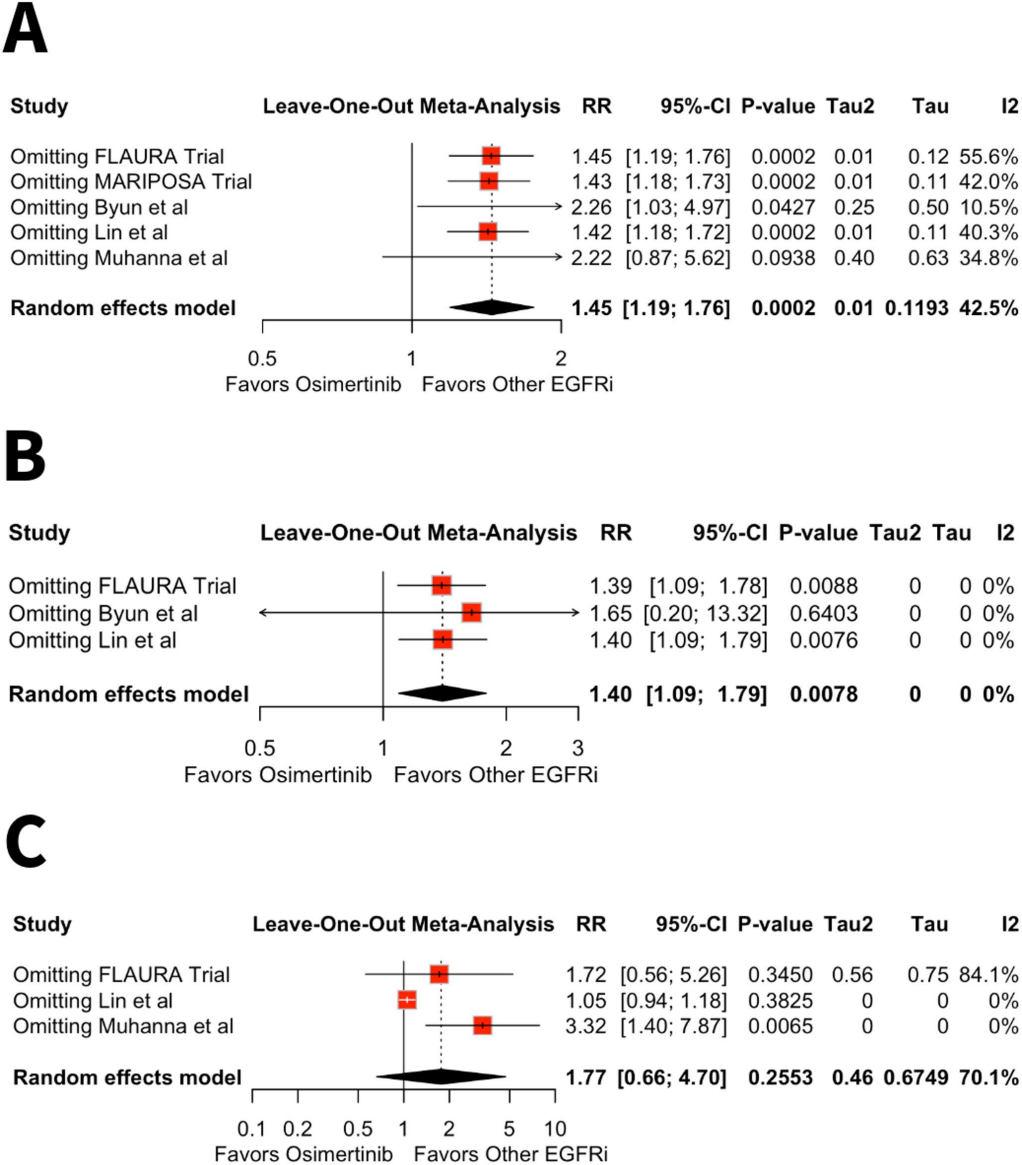
**A**-**C** Sensitivity analysis of HF **A**, MI **B** and arrhythmias **C** in patients receiving Osimertinib therapy compared to other EGFR inhibitors

The influence of large observational studies on pooled estimates was assessed through the prespecified exclusion of Byun et al. study. Following exclusion, Osimertinib therapy continued to remain significantly associated with increased risk of HF (RR 2.26, 95% CI 1.03–4.97, *p* = 0.04, I² = 10.5%) (Fig. 3A). In contrast, association between Osimertinib therapy and MI was attenuated and no longer statistically significant following the exclusion of Byun et al. (RR 1.65, 95% CI 0.20–13.32, *p* = 0.64, I² = 0%) (Fig. 3B).

Sensitivity analysis testing of arrhythmia using leave-one-out approach demonstrated no significant difference in the risk of arrhythmia following the exclusion of study by Lin et al. (RR 1.05, 95% CI 0.94–1.18, *p* = 0.38, I² = 0%). However, exclusion of the study by Muhanna et al. resulted in an increased risk of arrhythmia with Osimertinib compared to other EGFR inhibitors (RR 3.32, 95% CI 1.40–7.87, *p* = 0.0065, I² = 0%) (Fig. 3C).

Meta-regression analysis was conducted to assess whether demographic and clinical variables moderated the risk of HF. Among all covariates tested, smoking status was the only statistically significant moderator. Specifically, a history of smoking was associated with a significantly increased risk of HF (β = 6.98; 95% CI 1.08 to 12.88; *p* = 0.02, I² = 0%), indicating a consistent effect across studies and underscoring the robustness of this association (Supplemental S7). In contrast other co-variates including age, female sex, adenocarcinoma, White and Asian race were not statistically significant moderators of risk.

## Discussion

Our comprehensive meta-analysis provides the most up-to-date comparative analysis regarding Osimertinib’s cardiotoxicity profile compared to other EGFR inhibitors in EGFR-mutant NSCLC. Across the pooled analyses, Osimertinib therapy was associated with a higher risk of HF, decline in LVEF, and MI; however, the robustness of these associations differed by endpoint. Collectively, these findings provide quantitative context to cardiotoxicity concerns previously raised in case series, pharmacovigilance reports and cohort studies [15, 24–26].

Among the evaluated outcomes, the association between Osimertinib and HF risk remained directionally consistent across included studies and sensitivity analyses, supporting HF as a reproducible and clinically meaningful cardiotoxic signal rather than an isolated finding driven by a single data source. This finding is in line with increased risk of HF observed across other post hoc analyses of RCTs (24). In parallel, the effect size of LVEF decline is particularly concerning with a 3.10-fold increased risk. Although heterogeneity in imaging and reporting may limit causal inference, this finding aligns with the HF signal suggesting a meaningful susceptibility to increased risk of Osimertinib-associated systolic dysfunction in at least a subset of patients.

Accumulating preclinical and translational data provide biological plausibility to these observations [24, 25]. Osimertinib’s off target disruption of signaling pathways, including neuregulin-1/ErbB and downstream PI3K/AKT, can result in a state of impaired cardiac stress response, rendering cardiomyocytes vulnerable to dysfunction and death [24, 25, 27–31]. Histopathological findings from real-world cohorts including cardiomyocyte hypertrophy, lipofuscin deposition, and focal vacuolization further support a direct cellular mechanism underlying Osimertinib associated myocardial injury [32]. These convergent findings from multiple independent investigations explain the reproducible pattern of cardiac dysfunction observed across diverse clinical populations, including the decline in LVEF and the development of HF.

Recent real-world studies have also evaluated the efficacy and overall safety of Osimertinib in specific molecular subgroups. Priantti et al. evaluated Osimertinib in patients with uncommon EGFR mutations and reported favorable oncologic efficacy with an acceptable overall safety profile. However, this study did not prespecify cardiovascular endpoints or directly compare Osimertinib with other EGFR-TKIs. Therefore, although this study informs treatment efficacy and broad tolerability, it provides limited insight into cardiac risk differentials [33]. In contrast, the present meta-analysis specifically evaluates comparative cardiotoxicity and focuses on cardiotoxic events, thereby addressing a critical gap at the intersection of thoracic oncology and cardio-oncology.

In contrast to HF and decline in LVEF, association between Osimertinib and MI was less robust and demonstrated greater sensitivity to influential large registry-based databases. Although, pooled estimates suggested an increased risk of MI with Osimertinib compared to other EGFR inhibitors, this association was attenuated in pre-specified sensitivity analyses suggesting a more cautious, hypothesis-generating interpretation. Experimental models have shown that Osimertinib impairs PI3K/AKT signaling, reducing nitric oxide (NO) bioavailability resulting in endothelial dysfunction and increased vascular oxidative stress. In vulnerable cancer populations, often older and harboring significant cardiovascular (CV) comorbidities, these molecular effects likely translate to compromised vascular homeostasis and increased risk of accelerated atherosclerosis resulting in greater susceptibility to ischemic injury [26, 34–38]. These findings underscore the importance of prospective studies with adjudicated ischemic endpoints and detailed cardiovascular phenotyping to clarify the relationship between Osimertinib exposure and atherothrombotic risk.

Our pooled analysis did not demonstrate a statistically significant increase in arrhythmic risk with Osimertinib therapy. However, the totality of mechanistic and clinical evidence signals a genuine and relevant pro-arrhythmic potential for this agent. Electrophysiological studies using ex vivo and in vivo models have demonstrated that Osimertinib blocks multiple cardiac ion channels, specifically, the rapid delayed rectifier potassium current (hERG), the major cardiac sodium current (Nav1.5), and L-type calcium channels at concentrations achieved in patients [34]. Notably, the inhibition of hERG by Osimertinib (IC₅₀ ≈ 2.21 µM) leads to substantial prolongation of the QT interval, while concurrent Nav1.5 block (IC₅₀ ≈ 3.2 µM) slows conduction and increases dispersion of repolarization, further increasing the propensity for both reentrant and triggered ventricular arrhythmias. Clinical translations of these findings are well-documented. Across large RCTs, such as FLAURA and MARIPOSA, Osimertinib consistently presented higher rates of QTc prolongation compared to comparator EGFR-TKIs, 10–17% versus 4% respectively [7, 34, 35]. Furthermore, other real-world datasets and case series reported rare but life-threatening events, including cases of torsades de pointes and ventricular arrhythmias in patients with polypharmacy, electrolyte imbalance, or pre-existing conduction defects [26]. Importantly, these arrhythmic events are often reversible upon Osimertinib therapy discontinuation or dose adjustment, lending further credence to a reversible, pharmacodynamic mechanism rather than irreversible myocardial injury [25]. Finally, arrhythmic risk may be time-dependent, with early drug-related QT prolongation differing from later arrhythmias associated with cumulative exposure, electrolyte abnormalities, or progressive myocardial dysfunction. The significant heterogeneity in the arrhythmia pooled estimates likely reflects variability in comparator groups, outcome definitions based on broad ICD-based codes, ECG monitoring frequency and follow up duration. These factors likely underlie the sensitivity of pooled arrhythmia estimates to influential studies, such as Muhanna et al., whose exclusion resulted in a marked change in effect estimates. Collectively, these sources of heterogeneity limit the interpretability of pooled arrhythmia estimates and necessitate a more cautious interpretation of this outcome.

Our analysis did not reveal an increased risk of pericardial effusion with Osimertinib therapy, indicating that its cardiotoxicity profile likely does not include serosal inflammation or capillary leak, as is often seen with immune checkpoint inhibitors and some traditional chemotherapeutics [36]. This distinction reinforces mechanistic observations showing that Osimertinib’s main off-target cardiac effects are specific to myocardial and electrical injury without affecting pericardial or vascular serosa [34, 37].

Exploratory meta-regression suggested that among all clinical and demographic covariates examined, a history of smoking emerged as the sole statistically significant effect modifier of EGFR-TKI–associated risk of HF. Smoking is independently associated with impaired endothelial function, increased oxidative stress, and acceleration of subclinical and overt atherosclerosis largely driven by the generation of reactive oxygen species, decreased NO bioavailability, and chronic inflammatory signaling [38]. These processes may intersect with the PI3K/AKT and HER2/ErbB2 pathways, enhancing susceptibility to cardiotoxicity [17, 35, 39]. Emerging cardio-oncology research now suggests that these mechanisms may have synergistic or multiplicative effects when combined with EGFR inhibition, especially with chronic exposure [36, 39]. These mechanistic considerations provide biologic plausibility for smoking as a potential moderator of HF risk with Osimertinib. However, the underlying analyses are inherently exploratory and based on study-level covariates; detailed information on smoking intensity, duration, and cumulative exposure was not consistently available, and residual confounding by other lifestyle and comorbid conditions is likely.

Despite the overall low frequency of severe events, the magnitude of relative risk increase underscores the importance of vigilant cardiac monitoring. Our findings support current recommendations for baseline and periodic ECG and echocardiography in patients receiving Osimertinib, particularly those with pre-existing CV risk factors. Early detection and management of cardiac dysfunction may mitigate the risk of irreversible damage and allow for continued oncologic benefit from Osimertinib therapy. Consequently, routine pericardial imaging is not indicated unless clinical symptoms develop, with clinical focus better placed on surveillance for HF and arrhythmia.

## Limitations

Several limitations of this meta-analysis warrant careful considerations. The pooled estimates for HF and MI were substantially influenced by large population based observational cohorts, particularly the SEER-Medicare analysis reported by Byun et al. This study was restricted to patients aged 65 or older with continuous Medicare Part D coverage, limiting generalizability to younger patients and to those without Medicare-based insurance. Ascertainment of CV outcomes using claims may be subject to misclassifications, lacks standardized clinical adjudication, and does not capture detailed clinical information such as left ventricular function, biomarkers, or coronary anatomy. From an analytical standpoint, Byun et al. used inverse probability of treatment weighting without stabilized weights, a strategy that can inflate the effective sample size and artificially narrow confidence intervals, thereby giving an impression of greater precision [40]. Although extensive covariate adjustment was performed, residual confounding from unmeasured factors such as frailty, baseline cardiac function, smoking intensity, cancer stage and prior cardiotoxic therapies is possible and cannot be excluded.

Differences in survival and time-at-risk differences represent an additional source of potential bias in comparative cardiotoxicity assessment. Osimertinib improves progression-free and overall survival relative to earlier-generation EGFR-TKIs in advanced EGFR-mutant NSCLC, often resulting in longer treatment duration and extended follow-up [6–9]. Consequently, patients treated with Osimertinib may therefore have a longer window during which CV events can be detected, independent of any true difference in per-unit time risk resulting in an exposure-time or survival bias. Our meta-analyses cannot fully harmonize time-to-event distributions, adjust differential censoring or systematically evaluate cumulative dose or treatment duration. These constraints highlight the need for individual patient-level datasets and prospective cardio-oncology studies to more clearly distinguish drug-related cardiotoxicity from time-dependent detection effects.

Inclusion of non-randomized studies can introduce potential biases, including confounding, selection, and recall bias. Although random effects model and sensitivity analyses were used to partially account for between study heterogeneity and variability in study design, comparator agents, follow-up duration and outcome definitions. The use of aggregated study level data limited our ability to adjust for detailed patient-level confounders, such as baseline CV risk, prior cardiac history, performance status, and concomitant cardiotoxic or cardioprotective factors. This constraint also precludes assessments of cumulative dose and evaluation of competing risk, all of which are particularly relevant in oncology populations with high non-cardiovascular mortality. Furthermore, there was notable variability in Osimertinib dosing and duration of exposure across included studies. Finally, mild asymmetry in the funnel plot of HF raises the possibility of small-study or publication bias, although formal statistical testing was not undertaken because of the limited number of studies, consistent with methodological guidance from contemporary systematic review standards.

Taken together, these limitations indicate that the pooled estimates should be interpreted as reflective of association rather than definitive causal effects. This necessitates the need for prospective, adequately powered cardio-oncology studies with adjudicated cardiovascular endpoints, standardized imaging and echocardiographic surveillance and time-to-event analyses to more precisely characterize the CV safety profile of Osimertinib.

## Conclusion

Osimertinib therapy may be associated with higher risk of HF and LVEF decline compared with other EGFR-TKIs, with these findings representing the most consistent and reproducible cardiotoxic signals across available evidence. In contrast, association with MI and arrhythmias were less robust and sensitive to the inclusion of large observational cohorts and should be regarded as hypothesis-generating. No increased risk of pericardial effusion was observed. Although, the absolute incidence of CV events remains low, the increase in relative risk particularly for myocardial dysfunction supports a proactive approach to cardio-oncology care including baseline and periodic assessment of cardiac function and ECG monitoring especially in patients with pre-existing CV conditions or risk factors.

Given the potential for residual confounding, survival bias, and heterogeneous outcomes ascertainment, these findings should be interpreted as associative rather than causal and underscore the need for prospective, time-to-event-based studies with adjudicated cardiovascular endpoints. In the interim, close collaboration between cardiologist and oncologist remains essential to optimize the therapeutic benefit of Osimertinib while minimizing the potential risk of cardiotoxicity.

## Supplementary Information

Below is the link to the electronic supplementary material.

Supplementary Material 1.

## Data Availability

No datasets were generated or analysed during the current study.

## Abbreviations

EGFR: Epidermal growth factor receptor
NSCLC: Non–small-cell lung cancer
HF: Heart failure
MI: Myocardial infarction
LVEF: Left ventricular ejection fraction
QTc: Corrected QT interval
RR: Risk ratio
CI: Confidence intervals
FDA: Food and drug administration
TKI: Tyrosine kinase inhibitor
ErbB: Erythroblastic oncogene B
HER2: Human epidermal growth factor receptor 2
NRG1: Neuregulin-1
PI3K: Phosphoinositide 3-kinase
AKT: Protein kinase B
PRISMA: Preferred reporting items for systematic review and meta-analyses
AMSTAR 2: A measurement tool to assess systematic reviews 2
RCTs: Randomized controlled trials
RoB 2: Risk of bias 2
ROBINS-I: Risk of bias in non-randomized studies of interventions
CV: Cardiovascular
NO: Nitric oxide
IC₅₀: Half-maximal inhibitory concentration
ERK: Extracellular signal-regulated kinase
