# Supplementary material for: Cardiotoxic Effects of Osimertinib Compared to Other EGFR Inhibitors: A Systematic Review and Meta-Analysis
Source: Cardiovasc Toxicol. 2026 Mar 9;26(3):33. doi: 10.1007/s12012-026-10106-x (PMC12971819; doi:10.1007/s12012-026-10106-x)

**Supplementary Material**

**Supplemental Files Index:**

**Supplemental S1**: PRISMA 2020 guidelines checklist.

**Supplemental S2**: AMSTAR 2 guidelines checklist.

**Supplemental S3**: Research Question, PICO, MeSH, Keywords and Search Strategy.

**Supplemental S4**: Quality assessment using Risk of Bias 2 (RoB2) tool.

**Supplemental S5**: Quality assessment using Risk of Bias in Non-randomized Studies of Interventions (ROBINS-I) tool.

**Supplemental S6**: Funnel plot of heart failure in patients receiving Osimertinib therapy compared to other EGFR inhibitors.

**Supplemental S7**: Meta regression analysis of heart failure in patients receiving Osimertinib therapy compared to other EGFR inhibitors.

**Supplemental S8**: Bubble plots for meta-regression analysis of HF comparing Osimertinib to other EGFR inhibitors using age, female sex, and smoker as covariates.

**Supplemental S9**: Bubble plots for meta-regression analysis of HF comparing Osimertinib to other EGFR inhibitors using adenocarcinoma, White and Asian race as covariates.

**Supplemental S1: PRISMA 2020 guidelines checklist**

**Supplemental S2: AMSTAR 2 guidelines checklist**

**
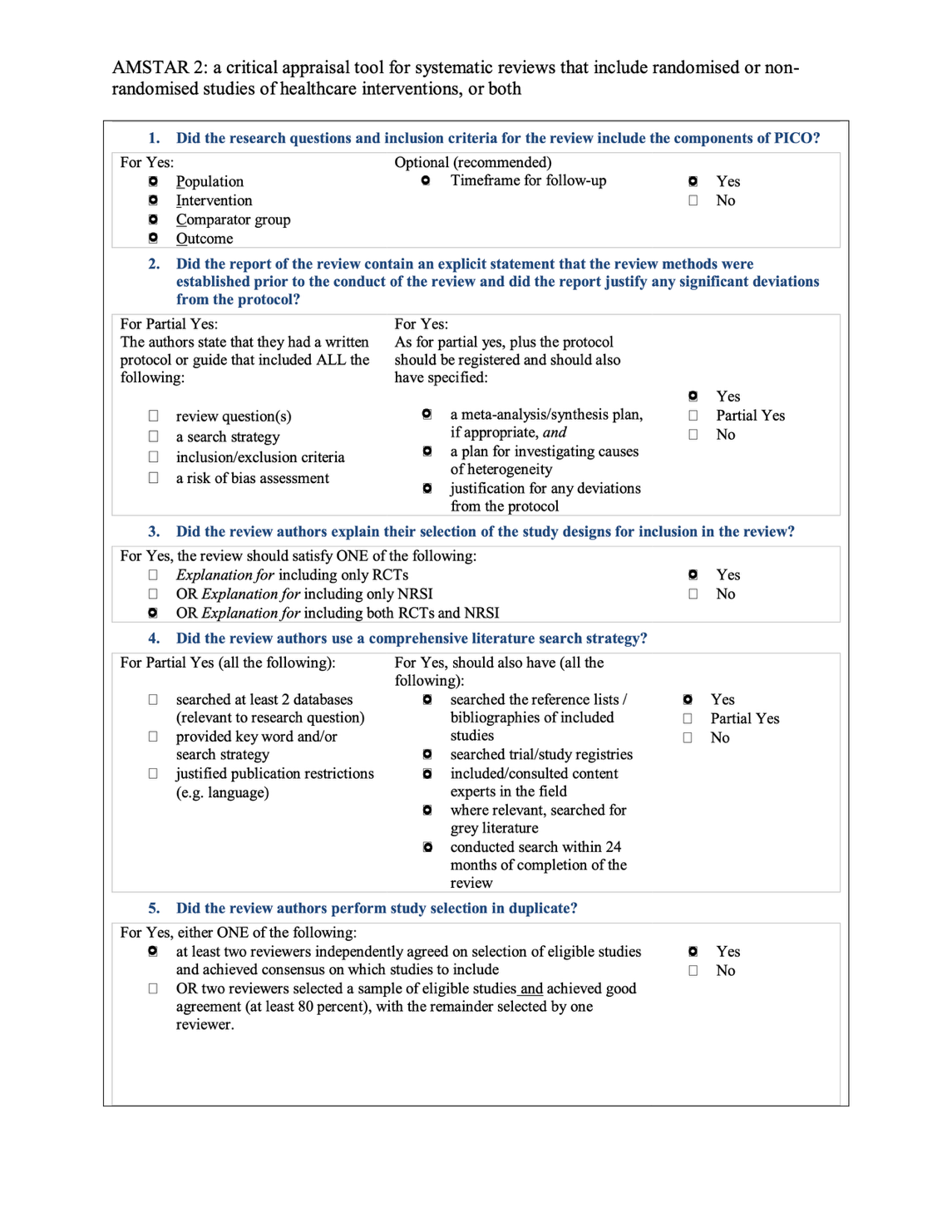
**


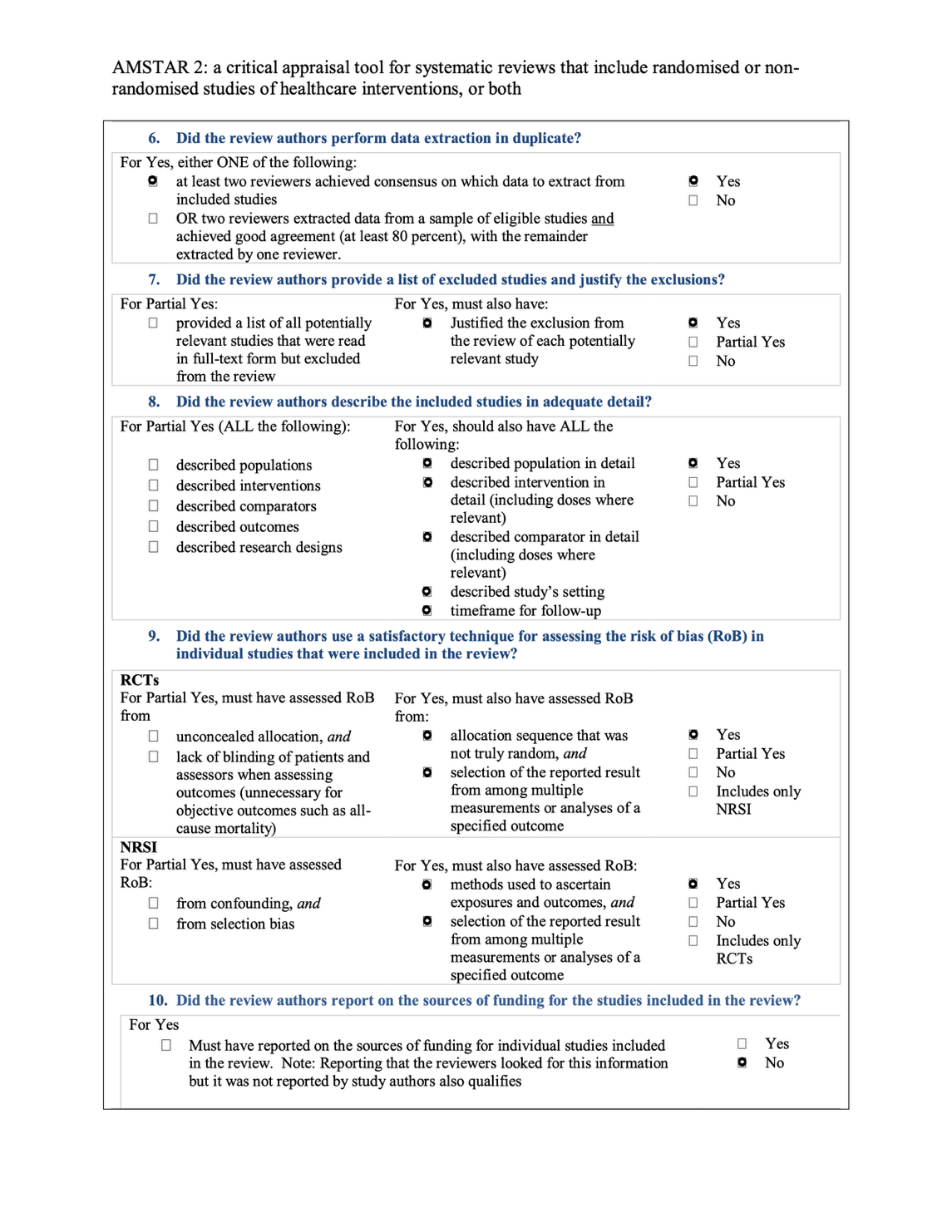


**
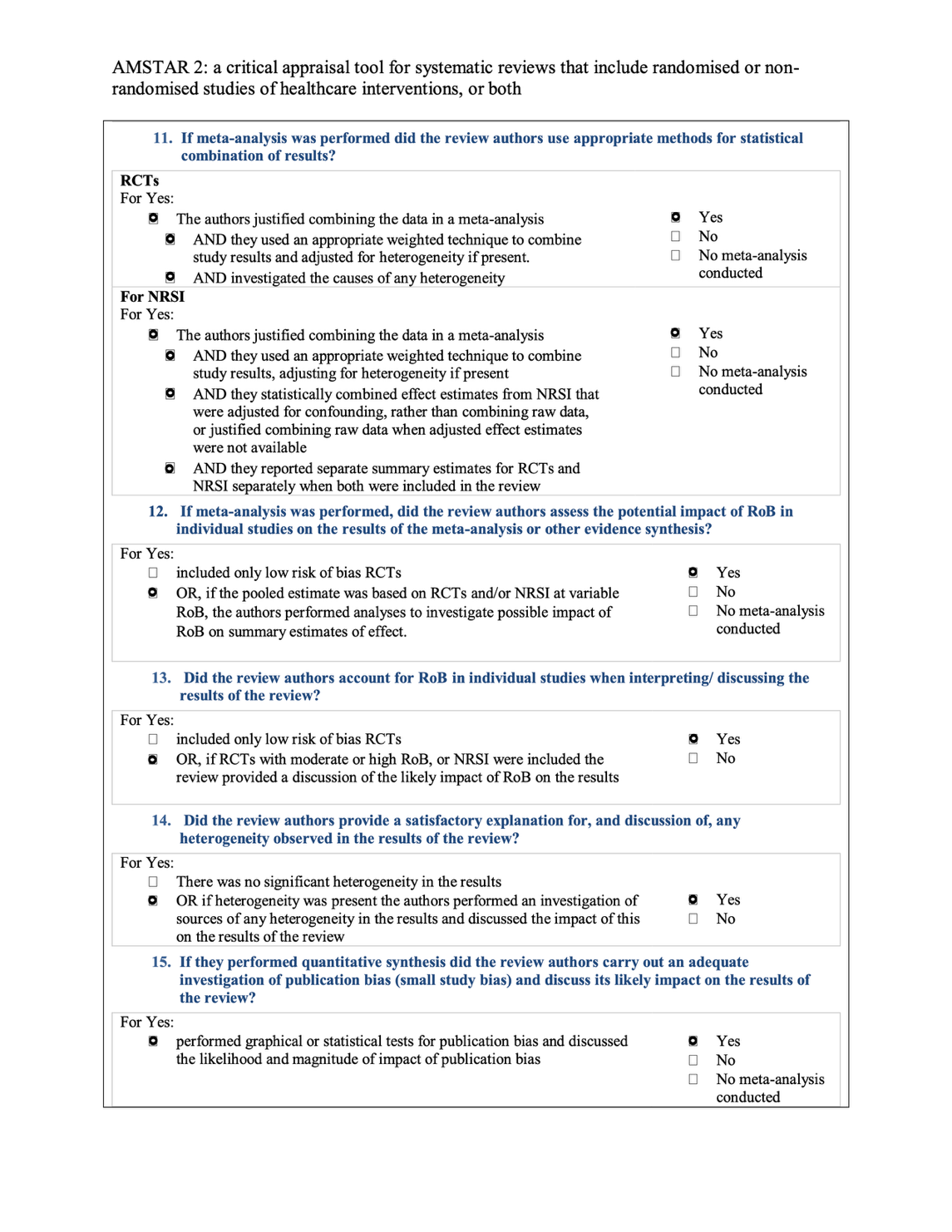
**

**
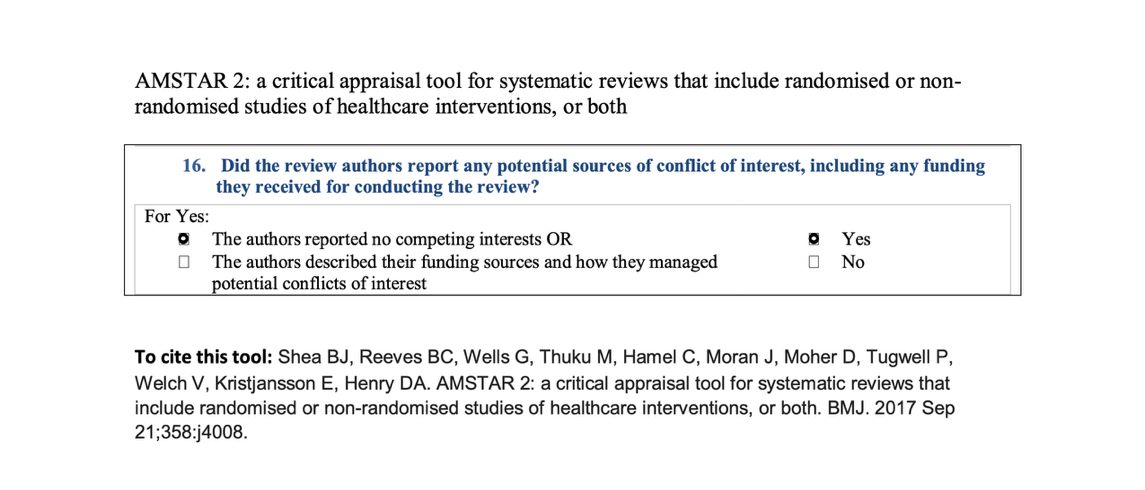
**

**Supplemental S3: Research Question, PICO, MeSH, Keywords and Search Strategy**

**Research Question:** Cardiotoxicity of Osimertinib Versus Other EGFR Inhibitors

**Population:** Non-Small Cell Lung Cancer

**Intervention:** Osimertinib therapy

**Comparator:** Other EGFR inhibitors

**Outcome:** HF, LVEF decline, MI, arrhythmias and MI

**Study Type:** Risk ratio (RR) for binary outcomes

**MeSH Terms and Key Words**

Non-Small Cell Lung Cancer

Osimertinib therapy

EGFR inhibitors

Heart Failure

Arrhythmias

**Search Strategy:**

| Database | Search strategy | Records retrieved |
| --- | --- | --- |
| PubMed | ("Osimertinib"[Mesh] OR osimertinib[tiab] OR "AZD9291"[tiab] OR EGFRi OR Epidermal Growth Factor Inhibitor OR tagrisso[tiab]) AND ("Cardiotoxicity"[Mesh] OR cardiotox*[tiab] OR "Heart Failure"[Mesh] OR "Ventricular Dysfunction, Left"[Mesh] OR "Cardiomyopathies"[Mesh] OR "Myocardial Infarction"[Mesh] OR "Ischemic Heart Disease"[Mesh] OR "Arrhythmias, Cardiac"[Mesh] OR "Atrial Fibrillation"[Mesh] OR "Long QT Syndrome"[Mesh] OR "QT interval"[tiab] OR "QTc"[tiab] OR "torsades"[tiab] OR "Sudden Cardiac Death"[Mesh] OR "cardiac adverse event*"[tiab] OR "cardiovascular adverse event*"[tiab] OR "major adverse cardiovascular event*"[tiab] OR MACE[tiab] OR myocarditis[tiab] OR pericarditis[tiab]) | 343 |
| Web of Science | (osimertinib OR "AZD9291" OR Tagrisso) AND ("epidermal growth factor receptor inhibitor*" OR "EGFR inhibitor*" OR "EGFR-TKI*" OR "EGFR TKI*" OR erlotinib OR gefitinib OR afatinib OR dacomitinib) AND (cardiotox* OR "cardiac toxicity" OR "cardiovascular toxicity" OR "heart failure" OR cardiomyopath* OR "myocardial infarction" OR "ischemic heart disease" OR arrhythmia* OR "atrial fibrillation" OR "QT interval" OR QTc OR torsades OR "sudden cardiac death" OR myocarditis OR pericarditis OR "cardiac adverse event*" OR "cardiovascular adverse event*" OR "major adverse cardiovascular event*" OR MACE) | 146 |
| Cochrane Library | (osimertinib OR AZD9291 OR Tagrisso) AND (erlotinib OR gefitinib OR afatinib OR dacomitinib) AND (lung OR "non small cell" OR NSCLC) AND ( cardiotox* OR "cardiac toxic*" OR "cardiovascular toxic*" OR cardiac OR cardiovascular OR toxicity OR safety OR "adverse event*" OR "heart failure" OR cardiomyopath* OR "left ventricular dysfunction" OR "myocardial infarction" OR "ischemic heart disease" OR ischem* OR arrhythmia* OR "atrial fibrillation" OR "long QT" OR "QT interval" OR QTc OR torsades OR "sudden cardiac death" OR myocarditis OR pericarditis OR "cardiac adverse event*" OR "cardiovascular adverse event*" OR "major adverse cardiovascular event*" OR MACE) | 112 |

**Supplemental S4: Risk of bias assessment using Risk of Bias 2 (RoB2) tool**


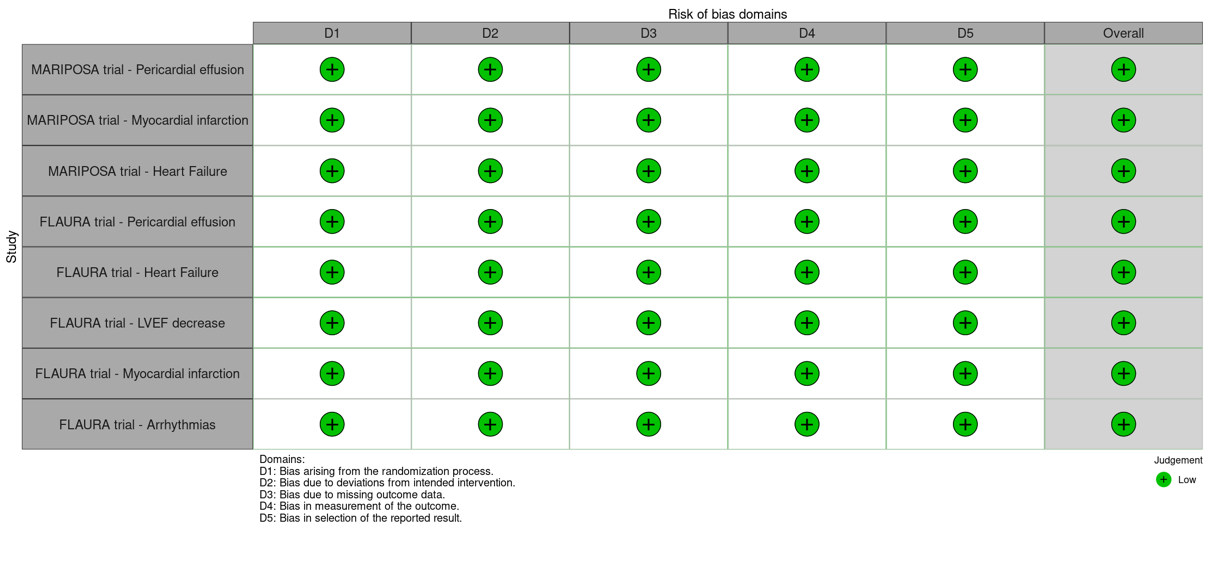


**Supplemental S5: Risk of bias assessment using Risk of Bias in Non-randomized Studies of Interventions (ROBINS-I) tool**

**
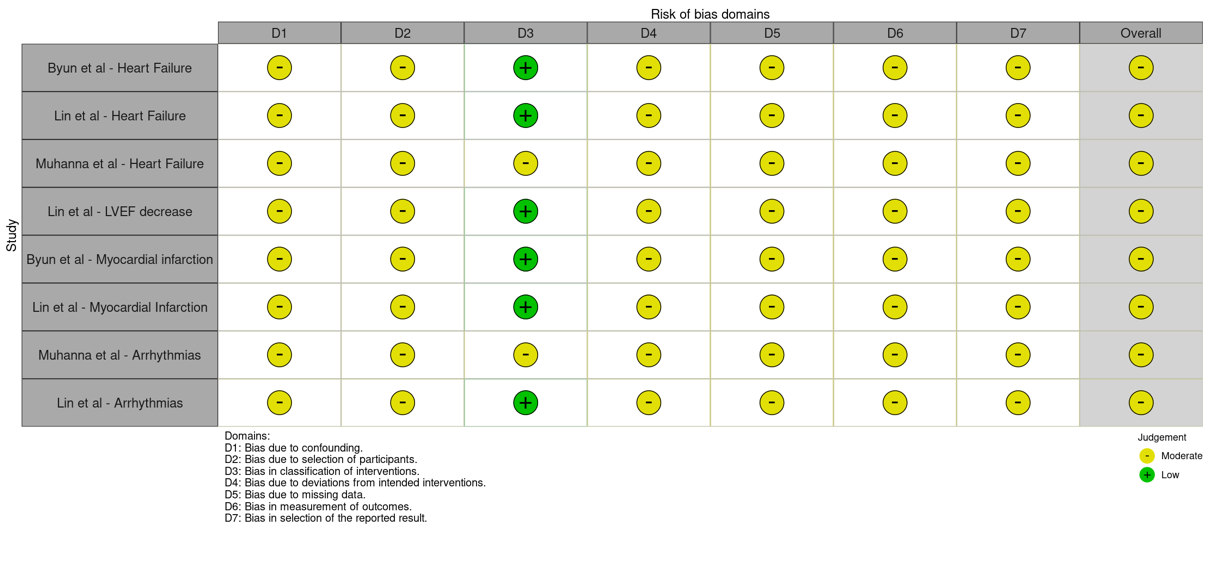
**

**Supplemental S6: Funnel plot of HF in patients receiving Osimertinib therapy compared to other EGFR inhibitors**

**
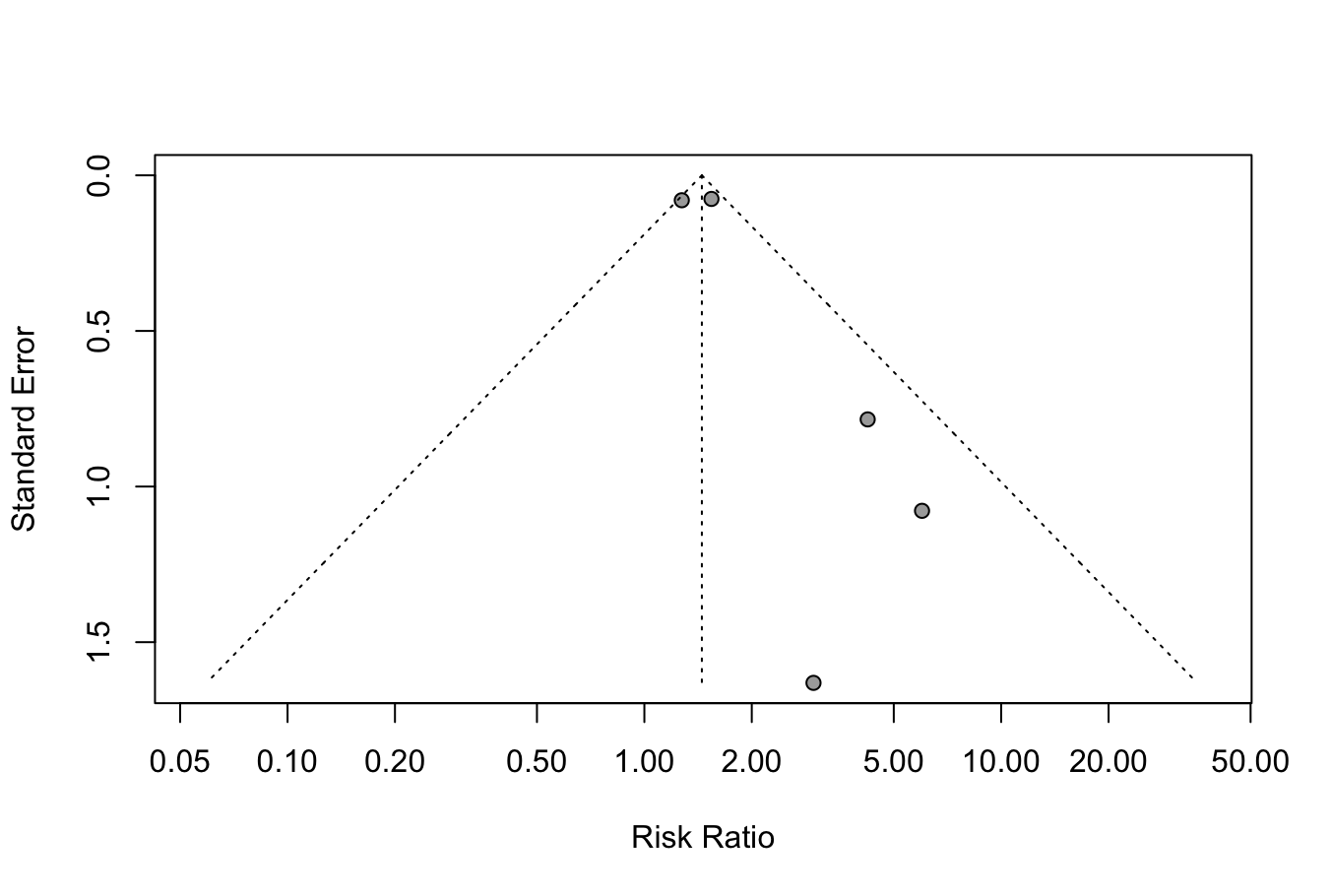
**

**Supplemental S7: Meta regression analysis of HF in patients receiving Osimertinib therapy compared to other EGFR inhibitors**

| Outcome | Co-Variate | Β-Coefficient | CI | p-value |
| --- | --- | --- | --- | --- |
| Heart Failure | Age | -0.05 | -0.14 - 0.04 | 0.28 |
|  | Female | -12.09 | -33.25 - 9.06 | 0.26 |
|  | Smoker | 6.98* | 1.08 - 12.88 | 0.02 |
|  | Adenocarcinoma | 35.29 | -13.76 - 84.33 | 0.16 |
|  | White | -2.54 | -8.44 - 3.36 | 0.40 |
|  | Asian | 2.70 | -2.83 - 8.24 | 0.34 |

**Supplemental S8: Bubble plots for meta-regression analysis of HF comparing Osimertinib to other EGFR inhibitors using age, female sex, and smoker as covariates**

**
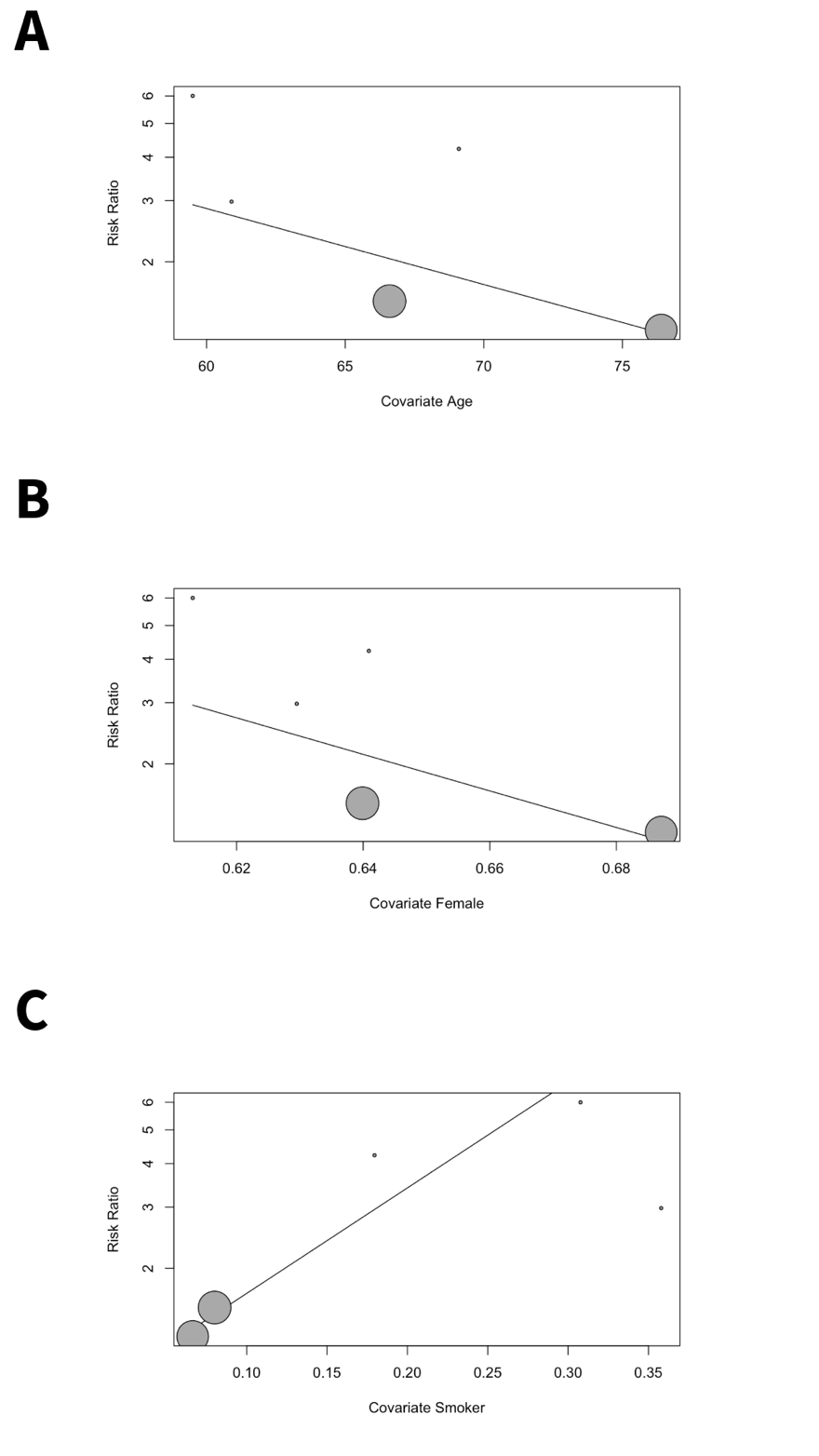
**

**Supplemental S9: Bubble plots for meta-regression analysis of HF comparing Osimertinib to other EGFR inhibitors using adenocarcinoma, White and Asian race as covariates**


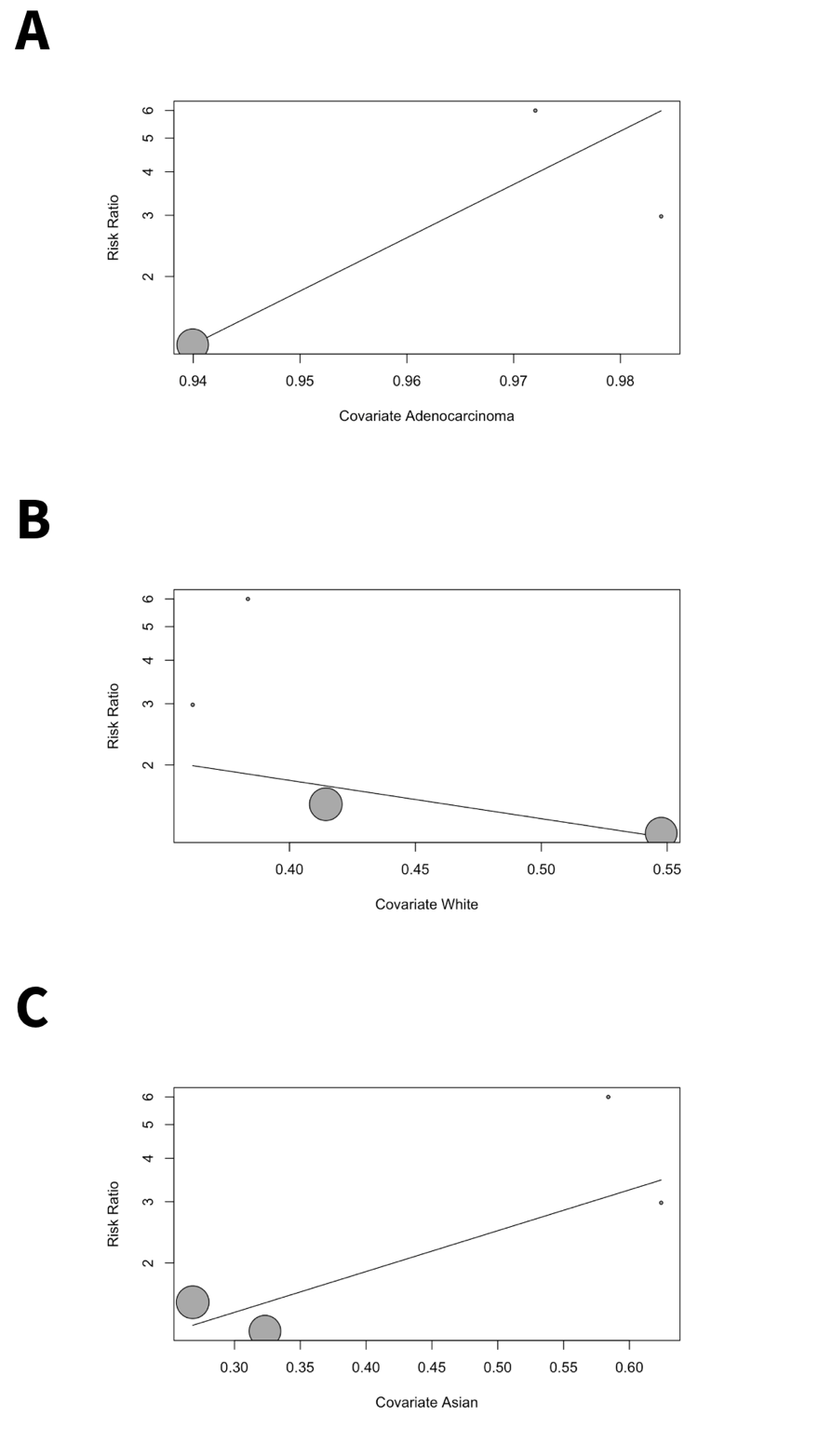

Supplement: Supplementary file 1 — Supplementary Material 1. [file 12012_2026_10106_MOESM1_ESM.docx]
